# Equine osteoarthritis: is intra-articular polyacrylamide gel more effective than corticosteroid in improving lameness?

**DOI:** 10.18849/ve.v7i1.413

**Published:** 2022-01-19

**Authors:** Constance Bowkett-Pritchard+

**Keywords:** EQUINE, OSTEOARTHRITIS, DEGENERATIVE JOINT DISEASE, INTRA-ARTICULAR, CORTICOSTEROID, TRIAMCINOLONE, METHYLPREDNISOLONE, POLYACRYLAMIDE

## Abstract

PICO question

In horses with naturally occurring osteoarthritis, is treatment with intra-articular polyacrylamide gel more likely to reduce the severity of clinical signs associated with lameness when compared to treatment with intra-articular corticosteroid?

Clinical bottom line

Category of research question

Treatment

The number and type of study designs reviewed

Twelve studies; four case series, three uncontrolled prospective studies, one non-blinded, non-randomised control trial, one non-blinded randomised control trial, two systematic reviews and one systematic review and meta-analysis

Strength of evidence

Weak

Outcomes reported

Studies examined: Clinical signs relating to lameness after use of corticosteroid or polyacrylamide gel to treat osteoarthritis; improvement in lameness and treatment success (including return to work in some papers)

Conclusion

It is not possible to recommend one treatment over the other given the absence of studies which provide direct comparison. This highlights the need for further controlled and comparative studies

## How to apply this evidence in practice

The application of evidence into practice should take into account multiple factors, not limited to: individual clinical expertise, patient’s circumstances and owners’ values, country, location or clinic where you work, the individual case in front of you, the availability of therapies and resources.

Knowledge Summaries are a resource to help reinforce or inform decision making. They do not override the responsibility or judgement of the practitioner to do what is best for the animal in their care.

## The evidence

There are no studies that directly compare intra-articular corticosteroid (CS) with polyacrylamide gel (PAAG) to treat equine osteoarthritis (OA). Therefore, studies that investigated either of the two treatments to manage naturally-occurring OA were examined. Twelve studies were identified, six investigating CS and six investigating PAAG. The evidence was generally of low quality, comprising largely of non-controlled studies. There were four case series, three uncontrolled prospective studies, one non-blinded, non-randomised control trial, one non-blinded randomised control trial, two systematic reviews, and one systematic review and meta-analysis. Both controlled trials investigated CS treatment although one was poorly designed and did not use numerical or statistical data to support its conclusions. Three case series reported use of CS and were of poor quality with variable treatment regimens used and subjective outcomes measured. A further case series reported intra-articular PAAG and was limited by subjective assessment, a small population, and lack of detail regarding previous therapeutic agents administered to the cases. Both systematic reviews (one CS and one PAAG) were limited by the lack of controlled trials. The systematic review and meta-analysis (PAAG), whilst using a thorough literature search, was also restricted by the lack of controlled trials and the heterogeneity of reported studies limited the statistical analysis that could be performed.

## Summary of the evidence

**van Pelt (1963):** 

Population:	Signalment: Age: 3.6 ±4 years (range 1.5–10 years). Sex: 12 stallions, nine mares, and seven geldings. Diagnoses based on history, clinical signs, arthrographs, synovial fluid analysis, a synovial membrane specimen, and bacteriology. Cattle were also studied but are not discussed here as this is outside the scope of the Knowledge Summary.
Sample size:	28 horses (45 joints) including 22 horses diagnosed with OA. Other pathologies included were: Osteochondritis dissecans (n = 1). Traumatic arthritis (n = 6). Synovial effusion of the tarsocrural joint (n = 12). Synovitis (n = 4) (presumably not related to the tarsocrural joint). Tenosynovitis (n = 2). Bursitis (n = 2).
Intervention details:	No systemic adrenocorticotrophic hormones, adrenocortical steroids or their synthetic analogues were administered prior to or during the course of intra-articular injections. Blood serum samples taken by jugular venipuncture for detection of alkaline phosphatase activity and serum sugar values (total reducing agents) immediately prior to intra-articular injection. Sugar values are not defined further by the authors. Synovial fluid obtained by arthrocentesis from radiocarpal, intercarpal, metacarpophalangeal, metatarsophalangeal, tarsocrural, femoropatellar, and scapulohumeral joints. In some instances, synovial fluid was also collected from the same joint in the opposite limb. Using the arthrocentesis needle, horses were injected with varying amounts (80–200 mg) of 6α methylprednisolone acetate (40 mg/cc) (Depo-Medrol®, the Upjohn Co., Kalamazoo, MI, USA). A single injection was made into 36 joints of 20 horses. A second intra-articular injection was made into eight tarsocrural joints of eight horses 46±14 days after the first injection: Five with chronic tarsal synovial effusion, two with traumatic arthritis, one with osteochondritis dissecans. A third intra-articular injection was made into the tarsocrural joint of one horse (with synovial effusion) 60 days after the second injection.
Study design:	Case series.
Outcome studied:	A combination of subjective and objective measures were used: Blood samples: Whole blood sugar value (total reducing agents). Serum sugar value (total reducing agents). Serum alkaline phosphatase activity. Synovial fluid: Synovial sugar levels (total reducing agents). Synovial alkaline phosphatase activity. Viscosity – using a viscosimeter. Mucin clot test. Total and differential leukocyte counts. Total erythrocyte count. Clinical Response – the following parameters are described in the results section: Resolution of effusion. Improvement in gait. Relief from pain. Alleviation of inflammation.
Main Findings (relevant to PICO question)	‘Excellent’ clinical response was observed. Relief from pain and inflammation was evident within 24 hours after intra-articular injection. Maximum improvement occurred within 72 hours. No horses suffering from OA required a repeat injection.
Limitations:	Signalment lacking detail; no information given regarding the breed or use of the horses involved. No data reported regarding the duration or severity of pathology prior to injection. No inclusion or exclusion criteria mentioned. Methods used to diagnose OA not explicit; there is no mention of diagnostic anaethesia. Variable doses of CS used between horses. Timings of repeat synoviocentesis and intra-articular medications are inconsistent (although this did not involve horses diagnosed with OA). The outcomes used to measure clinical response are vague and subjective. Small sample size. No control group. Synovial fluid analysis was only performed on horses which underwent multiple intra-articular injections ― this could have led to bias and considerably reduces the sample size. Results regarding clinical response are not correlated with the severity of pathology diagnosed. No numerical data given regarding the individuals which responded. Reporting is inconsistent – the authors say a second intra-articular injection was performed in eight tibiotarsal (tarsocrural) joints in eight horses but in Table 5 there is a discrepancy; ‘8 tibiotarsal joints of 7 horses’. No definition given regarding ‘non-responders’. No long-term outcomes were measured.

**van Pelt (1970):** 

Population:	Horses affected by arthritis based on anamneses, clinical signs, physical and radiographic examination, blood and synovial fluid analysis. Signalment: Age: 9 months to 7 years. Sex: Three geldings, two stallions, two mares, and one filly. Breed: Four Standardbreds, one Thoroughbred, one Shetland pony, one Appaloosa, one Canadian Hunter. Diagnosis: number of horses affected: Traumatic arthritis: Two. Synovial effusion: One. Osteochondritis dissecans: One. Degenerative joint disease: Four tarsi. This will be the focus in accordance with the Knowledge Summary PICO question. The tarsus was affected bilaterally (n = 2) or unilaterally (n = 2). Tarsocrural and proximal intertarsal joints were affected radiographically – the joint affected was not specified for all horses. Age: 4–7 years. Sex: One stallion, one gelding, and two mares. Breed: Three Standardbred (used for racing), one Canadian Hunter. Horses with intractable forms of degenerative joint disease, traumatic arthritis with internal derangement of the joint, and septic arthritis were excluded. For part of the study five ‘healthy’ controls were used: Age: 3–15 years. Sex: Geldings. Breed: Thoroughbred.
Sample size:	13 horses Cases horses: Eight, 11 joints including: Four horses with degenerative joint disease, six joints. Control horses: Five.
Intervention details:	Blood samples for determination of serum sugar content (total reducing substances) were collected from jugular vein of all horses before arthrocentesis. Samples of synovial fluid were aspirated from affected joints in clinical cases (prior to injection). from tarsal joints (the particular joint sampled is not specified) in control horses. Cases received intra-articular injection of preparation containing betamethasone disodium phosphate (3 mg/ml) and betamethasone acetate (12 mg/ml) (Betavet Soluspan, Schering Corporation, Inc., Bloomfield, NJ, USA). Dose was 1.5–10 ml depending on joint size, severity and duration of joint disease. The joints injected were not specified. Repeat intra-articular injections were performed ‘as the need arose’. Serial synovial fluid analysis was only performed on two horses which required repeated intra-articular injection (one with bilateral tarsal synovial effusion and one with chronic osteochondritis dissecans).
Study design:	Case series (with regard to clinical outcomes).
Outcome studied:	Objective and subjective parameters were used. Serum: Serum sugar content (total reducing substances). Synovial fluid: Relative viscosity. Mucinous precipitate quality. Hyaluronic acid concentration. Sugar content (total reducing substances). Protein content. Albumin:globulin ratio. Enzyme activity: alkaline phosphatase, acid phosphatase, lactic dehydrogenase, aldolase, glutamic oxaloacetic transaminase and glutamic pyruvic transaminase. Total and differential leukocyte count. Total erythrocyte count. Clinical assessment: Return to normal gait. Relief from pain and joint stiffness. Range of joint motion. Reduced distension of the joint capsule. Lack of tenderness on palpation.
Main Findings (relevant to PICO question)	Six horses, including those with degenerative joint disease, ‘responded’ to a single intra-articular injection. Symptomatic relief was apparent in 24–48 hours. The three Standardbred horses returned to active training following a short period (undefined) of stall rest. The authors describe ‘permanent’ remission of clinical signs of joint disease as manifested by improvement or return to normal gait.
Limitations:	No mention of diagnostic anaethesia to diagnose arthritis. No case definitions given for the different diagnoses. Small sample size. Clinical information omitted e.g. degree of lameness before and after joint medication, duration of lameness prior to medication, prior or concurrent treatments. Variable dose of CS was used. Methodology lacked detail, e.g. no description of how joint capsule distension and range of motion were measured and how ‘normal range of motion’ was defined. Post-medication exercise protocol was not made explicit and appeared to vary. Clinical outcomes were vague and subjective. Observers were not blinded. No control group to facilitate comparison of improvement in lameness (the control group was ‘healthy’ and only used for synovial fluid analysis). There was no follow-up provided, including whether horses were able to return to their intended use and the interval between repeated joint medications.

**van Pelt (1971):** 

Population:	Horses affected with joint disease. Diagnosis was based on anamneses, clinical signs, physical and radiographic examination, serum and synovial effusion analysis and results of bacterial culture of synovial effusion samples. Signalment: Age: 4 months to 9 years old. Sex: Six fillies, four colts, three geldings, and one stallion. Breed: Three Arabians, seven Standardbreds, one Quarter Horse, one Morgan, one Tennessee Walking Horse, and one Thoroughbred. Diagnosis: number of horses and joint affected: Tarsal synovial effusion: Seven, tarsocrural joint. Postoperative arthritis: One, tarsocrural joint. Traumatic arthritis: One, metacarpophalangeal joint. Degenerative joint disease: Four, tarsocrural joint; one, radiocarpal joint. This will be the focus in accordance with the Knowledge Summary PICO question. Radiographic evidence of OA included narrowed joint spaces, marginal osteophytes, and an increase in subchondral bone density. Age: 4–9 years. Sex: One colt, one stallion, three geldings. Breed: Standardbred. Use: Racing. Bilateral disease: Two (both tarsocrural joints);a further horse appears to be bilaterally affected but both joints are described as left tarsocrural. This is thought to be an error. Unilateral disease: Three (two tarsocrural and one radiocarpal). Clinical signs: ‘intermittent or persistent’ lameness described. Two horses had history of prior intra-articular injection prior to inclusion in the study. Healthy adult horses acted as a control population.
Sample size:	14 horses with joint disease: Five with degenerative joint disease (OA). Seven with synovial effusion (tarsocrural joint). One with traumatic arthritis. One with post-operative arthritis. Seven control horses.
Intervention details:	Blood samples were collected from jugular vein to determine serum sugar content (total reducing substances). Synovial fluid was aspirated from joints of horses affected with joint disease and control horses (radiocarpal/intercarpal and tarsocrural joints). Intra-articular injection with flumethasone (Anaprime Suspension Veterinary (2 mg/ml); Animal Health Division, Syntex Laboratories, Inc., Palo Alto, CA, USA) performed ― for degenerative joint disease group the dose ranged from 4–10 mg. Larger amounts injected into tarsocrural joint compared to the radiocarpal joint. Two horses received a second injection 7–9 days following the first injection. Synovial fluid samples aspirated each time arthrocentesis was performed: 2–30 (mean 10.7) days after initial steroid injection. Therefore, not all horses underwent repeat synovial fluid analysis.
Study design:	Case series (regarding clinical outcomes for degenerative joint disease).
Outcome studied:	Subjective and objective outcomes were studied. Serum: Serum sugar content (total reducing substances). Synovial fluid: Relative viscosity. Mucinous precipitate quality. Sugar content (total reducing substances). Total protein. Albumin and globulin content. Ratio of albumin : globulin. Total erythrocyte count. Total and differential leukocyte counts. Bacterial culture (performed in 3 horses, none with degenerative joint disease). Clinical response: Methods used to measure this were not described in detail. Normal gait, joint motion, capsular distension and pain on manipulation are described in the results section. Beneficial and adverse systemic side effects: Methods to assess this were not described.
Main Findings (relevant to PICO question)	Clinical response (degenerative joint disease group): Return to normal gait in three horses 24–48 hours after injection: Normal range of joint motion, reduced capsular distension and lack of pain on manipulation are described. One of these horses received a second injection 7 days after the first. One horse showed a poor response (euthanased 7 days after injection at owner’s request). One horse (radiocarpal joint affected) showed a minor improvement in gait – this horse received phenylbutazone (2 g) (Butazolidin Veterinary; Jensen-Salsbery Laboratories, Kansas City, MO, USA) daily for 13 days and a second flumethasone injection after 9 days but no further improvement is described.
Limitations:	Small sample size. Discrepancy regarding the control horses – it is unclear if the radiocarpal or intercarpal joint was sampled; both are mentioned. Unclear if one horse in degenerative joint disease group was unilaterally or bilaterally affected. No mention of diagnostic anaethesia in lameness investigation. No case definitions given for the different diagnoses. Clinical information omitted e.g. degree of lameness before and after joint medication, duration of lameness prior to medication, details of prior treatments. No lame control group was used to compare the clinical effects of intra-articular flumethasone. Synovial fluid analysis was not consistent between horses. Varying doses of CS were used. The number and timing of (repeat) joint injections varied. Subsequent medications were added in some cases after a failure to respond adequately to the intra-articular CS (this occurred in one case in the degenerative joint disease group). No randomisation of treatments. No description of management post-medication is given. Observers were not blinded. Measurements regarding clinical outcomes were vague and subjective. No information on whether treated horses were able to return to their previous use. No long-term follow-up reported.

**Janssen (2012):** 

Population:	Horses treated at the Veterinary Clinic for Horses, Lüsche, Germany from 2008–2011. Age: 10 years (average), range 4–14 years. Sex: Nine geldings, two stallions, one mare. Breed: 11 Warmbloods, one pony. Use: 12 showjumping. Inclusion criteria: Forelimb lameness of at least 3 months’ duration. Osteoarthritis in either one or both forelimb distal interphalangeal joints (DIPJs). Affected joints treated intra-articularly at least twice with hyaluronic acid, CS and or autologous conditioned serum. MRI examination of the forelimbs with at least two of the following findings: Significant joint effusion. Subchondral bone changes. Cartilage damage. Bone cyst involving the joint. Increased fat-suppressed signal. Exclusion criteria: Horses with OA as a result of joint infection. Horses with skin diseases related to the DIPJ injection site. Additional MRI findings considered important, e.g. desmitis of the collateral ligament, tendinopathy of the deep flexor tendon. Horses that had undergone previous DIPJ arthroscopy.
Sample size:	12 horses.
Intervention details:	Each case was assessed for lameness in walk and trot in a straight line and on a circle on hard and soft surfaces. Lameness was graded using American Association of Equine Practitioners [AAEP] scale. In one horse with bilateral lameness, only the lamest limb was considered. The DIPJ was assessed for effusion using a grading system. Diagnostic anaesthesia: Lameness significantly improved to a palmar digital nerve block in all horses. Lameness improved by at least 50% after 1 minute following anaesthesia of the DIPJ with 5 ml mepivacaine (Scandicaine, AstraZeneca). Diagnostic imaging: Lateromedial and upright pedal radiographic views were obtained of the DIPJ in all horses. Magnetic resonance imaging (MRI) (0.27T Hallmarq Veterinary Imaging, Guildford, UK) of both front feet performed under standing sedation in all horses. Images were reviewed by an experienced observer. Therapeutic intervention: 1 ml of polyacrylamide gel (PAAG) (ArthramidVet, Contura International A/S, DK-2860 Søborg, Denmark) was injected into the DIPJ under sterile conditions. Horses were rested for 5 days then exercised for 2 weeks (two 20–minute walk sessions per day). Horses then ridden at walk and trot for one week. After the first follow-up (1 month) all horses had ‘light work’ for one month and the workload was then adjusted to the individual horse. Follow-up: Horses were reassessed at 1 and 6 months after PAAG injection.
Study design:	Retrospective case series.
Outcome studied:	Degree of lameness and joint effusion were assessed. Return to work was also mentioned in the results section.
Main Findings (relevant to PICO question)	Lameness: Average initial lameness score was 1.8 (range 1–3). At 1 month, the average lameness score was 0.8 (range 0–2): Four horses were sound, seven horses had improved (including the sound horses), four horses had an unchanged lameness, one horse had deteriorated. All horses showed a mild joint effusion. At 6 months, the average lameness score was 0.3 (range 0–1). Eight horses were sound, two horses were improved, and two were unchanged (one of these two subsequently had a palmar digital neurectomy performed). 8/12 (67%) of horses could return to show jumping. No adverse effects were seen.
Limitations:	The experience of the observer who assessed the radiographic and MR images is not made explicit. No grading system regarding severity of radiographic or MRI findings was described. However in the results section radiographic mild, moderate and severe OA is described – these are not defined by the authors. No objective lameness assessment included. No defined time period between previous unsuccessful treatments and PAAG injection. No detail regarding previous treatments administered to each horse. Limited detail regarding the presence of a clinically evident DIPJ effusion before and after treatment: Two horses had a severe joint effusion prior to treatment. On MRI all horses had increased DIPJ effusion. At 1-month follow-up, the authors state that all horses showed a mild effusion however only two underwent repeat MRI. Small population of horses. No control group. There is no mention of blinding of observers. Lack of detail describing the exercise regime after the first follow-up ― it was not uniform between horses. No statistical analysis performed ― likely due to small case numbers. Imaging findings not correlated to changes in lameness grade ― likely due to small case numbers. Follow-up limited to 6 months.

**Nicpoń (2013):** 

Population:	Horses diagnosed with early symptoms of bone spavin based on clinical signs and diagnostic anaethesia: Age: 8–14 years. Lameness: All horses described as severely lame.
Sample size:	16 horses.
Intervention details:	Research group – 10 horses: Cells were collected from the tail base, isolated according to Grzesiak et al. (2011) and cultured in vitro. Cells were suspended in sterile normal saline for infusion (concentration 5 x 10^6^ cells/ml). Intra-articular injection of 1 ml autologous adipose-derived mesenchymal stem cells. Comparison group – three horses: Intra-articular injection of 1 ml of betamethasone (concentration not given, product not listed). Control group – three horses: Restricted movement – exact protocol not described. Clinical evaluation: days 0, 30, 60, 90, and 180 after injection. Synovial fluid assessment: days 0, 90, and 180 after injection. Scintigraphy: days 0, 90, and 180 post-injection in two horses per group.
Study design:	Non-randomised non-blinded control trial.
Outcome studied:	Mostly subjective variables were studied: Lameness (no method or scale described). Synovial fluid analysis – measured parameters were not explicit; the following are mentioned in the results: inflammatory cell number – neutrophil number is also mentioned suggesting a differential count; total protein; viscosity; colour; turbidity. Scintigraphy: ‘Concentration of radionuclide in the tarsal joint’ is described.
Main Findings (relevant to PICO question)	Lameness: In the comparison (betamethasone-treated) group significant improvement and no signs of lameness were seen at 30, 60, and 90 days post injection. The research (stem cell-treated) group showed no change in lameness after 30 days but there was a great decrease after 60 days and no lameness at 90 and 180 days. On day 180 an increase in lameness in the comparison (betamethasone-treated) group was seen. In the control (rest only) group the degree of lameness did not change between 0 and 90 days; at 180 days there was a slight decrease in lameness.
Limitations:	Small sample size. Signalment not reported in detail (breed, sex, degree of lameness, use). Diagnostic imaging findings were not used as criteria for inclusion in the study ― horses may have had pathology other than bone spavin. No inclusion or exclusion criteria reported. Poorly detailed method. Prior or concurrent treatments were not mentioned. Exercise regimes for research and comparison groups were not mentioned and could have affected the outcome; confinement may have had a negative effect on the control group outcome. Variables measured were vague and often subjective. No randomisation of treatments. Observers not blinded. Reporting methods were extremely subjective with no individual data, numerical data or statistics included. Duration of follow-up was fairly short.

**Tnibar (2015):** 

Population:	Client-owned horses with OA in one joint (metacarpo(tarso)phalangeal or one of the carpal joints) that presented to two German and three Danish equine hospitals from October 2010 to February 2014: Age: Mean 9.4 years (range 2–15). Sex: Male, gelding, female (numbers not given). Breed: 30 Warmbloods, eight racing breeds, and five ‘other’. Use: 15 dressage, 13 jumping, eight racing, seven ‘other’. Diagnosis of OA was based on clinical evaluation, intra-articular analgesia (10 ml local anaesthetic per joint and examination 10 minutes after injection) and radiography. Exclusion criteria: Horses with lameness localised in more than one joint. Osteoarthritis secondary to joint infection. Horses that had undergone surgery of this joint less than 3 months preceding the study. Horses treated with any other anti-arthritic treatment administered to the affected joint less than 2 months preceding the study. Horses that received any additional anti-arthritic treatment or underwent surgery during the study period.
Sample size:	43 horses.
Intervention details:	On day 0 all horses were injected with 2 ml of polyacrylamide gel ((PAAG) Arthramid Vet, Contura International A/S, DK-2860 Søborg, Denmark) into the affected joint. Post treatment, horses were rested for the first two weeks with 10–15 minutes hand walking per day. For the next 2 weeks, all horses hand walked for 20–30 minutes per day or small paddock turnout. Horses examined at 1, 3, 6, 12, and 24 months post-treatment by clinicians different from the one who had originally examined and treated the horse: This second examiner was unaware of the identity of the horse and whether the horses were treated or not. 41, 26, and 40 horses of the total cohort (n = 43) were examined at 1, 3, and 24 months respectively. If horses were non-lame one month after treatment, they progressively resumed normal activity. All horses received only one injection of PAAG during the study.
Study design:	Prospective non-comparative blinded study.
Outcome studied:	Subjective variables were measured: Lameness grade (Ross, 2003). Joint effusion grade. Radiographic grading of OA (van Hoogmoed et al., 2003): baseline value only. Owner satisfaction (graded).
Main Findings (relevant to PICO question)	Lameness: Significant increase in the proportion of non-lame horses between baseline and 1 month, then a steady (non-significant) increase between 3–6 months followed by a stabilization in the proportion of non-lame horses between 6 and 24 months. 59%, 69%, 79%, 81% and 82.5% of horses non-lame at 1, 3, 6, 12, and 24 months respectively irrespective of baseline lameness grade. Of horses with a baseline grade 1 lameness (n=11), 3 showed worsening of the lameness grade at 3 (1/6 horses), 12 (2/11 horses) and 24 months (3/10 horses). No lameness worsening was observed in horses with baseline grade 2, 3, and 4. There was a highly significant reduction of lameness grade after baseline for all grades. Joint effusion: Joint effusion score decreased significantly over time.
Limitations:	No control group. Small sample size. Signalment incomplete. Variables measured were subjective. Use of multiple assessors may have led to inconsistency in the application of the grading systems. The repeatability of application of the radiographic grading system was not assessed. Exercise regimes were not standardised between horses after 1 month. Not all horses were examined at each follow-up time.

**de Grauw (2016):** 

Population:	Client-owned horses and ponies more than 2 years old presenting to 13 Dutch veterinary clinics between May 2011 and May 2012 with: Lameness at least grade 2 on a 0–5 scale (Ross, 2003). Lameness localised to one limb and one joint (distal interphalangeal, metacarpo(tarso)phalangeal, middle carpal or antebrachiocarpal). A positive response to intra-articular anaesthesia (defined as at least 50% visible improvement in lameness within 5 minutes after injection of local anaesthetic). The authors note that many horses underwent radiography and ultrasonography but imaging was not used as an inclusion criterion. Signalment: Age: Median 13 years (intervention A) and 11 years (intervention B); range 4–20 years. Use: Recreation 14 (A), 20 (B); sports 26 (A), 19 (B); breeding 1 (A), 0 (B). Exclusion criteria: Intra-articular injection in the same joint within 4 weeks prior to screening. Contraindications for intra-articular medication with CSs or severe comorbidity including joint infection, intra-articular fractures or fissures or laminitis. Lameness occurring in another limb between inclusion and study completion. Receipt of medication treating the musculoskeletal system between study inclusion and 3 week follow-up e.g. non-steroidal anti-inflammatories, polysulphated glycosaminoglycans and nutraceuticals.
Sample size:	80 horses.
Intervention details:	Horses randomly allocated into two treatment groups. Intervention A (triamcinolone (TA) Kenacort-A10, Bristol-Myers Squibb, Woerden, the Netherlands): 12 mg TA via intra-articular injection (n = 41 horses). Intervention B (TA + hyaluronic acid (HA) Hylartil vet, Zoetis BV, Capelle a/d IJssel, the Netherlands): 12 mg TA with 20 mg high molecular weight HA via intra-articular injection (n = 39 horses). Post-injection, all horses were box-rested on the day of treatment then underwent controlled walking exercise for 3 weeks. All horses re-evaluated at 3 weeks post-injection by the same veterinarian who performed the treatment. Depending on evaluation horses returned to work according to the veterinarian’s recommendations. Further treatment was initiated if lameness persistent according to the clinician’s judgement. Owners of surviving horses (n = 78) surveyed at 3 months post injection by the consulting veterinarian. 10 patients excluded from analysis at 3 months: Three horses in TA group and five in TA+HA group due to protocol violations. One horse from each group not alive at 3 months. Adverse events were recorded in an online database whenever an owner contacted the veterinarian with concerns possibly related to treatment any time between study inclusion and completion.
Study design:	Randomised non-blinded control trial.
Outcome studied:	Assessment was subjective. Variables measured: Lameness score. Joint effusion score. Primary outcome: Clinical success rate: proportion of horses that showed ≥ 2 grades reduction of lameness at 3 weeks compared to initial lameness score. Secondary outcome: Absolute reduction in lameness and effusion scores at 3 weeks compared to baseline. Proportion of horses that had returned to their previous level of performance at 3 months.
Main Findings (relevant to PICO question)	At 3 weeks TA group had significantly more cases classed as treatment success (36/41 (87.8%) vs 25/39 (64.1%) in TA+HA group). Lameness and effusion scores were significantly lower at 3 weeks compared to baseline for both groups. At 3 months 19/37 (51.4%) (TA) and 16/33 (48.5%) (TA+HA) of horses had returned to their previous level of performance.
Limitations:	Diagnostic imaging was not used as an inclusion criterion. Some signalment details were unreported (breed, sex). Subjective parameters were measured. Multicentre nature of the study may have meant that assessments were not repeatable. Observers and owners were not blinded at evaluations which may have introduced bias. Use of a controlled exercise programme after joint injection may have aided clinical improvement. Use of medication aimed at the musculoskeletal system permitted between 3 weeks and 3 months of follow-up. Use of owner-reported outcomes lessens the reliability of the results at 3 months. Limited follow up period of 3 months.

**McClure (2017):** 

Population:	Horses more than 2 years old and less than 636 kg presenting to ten United States veterinary clinics with: Lameness from a single OA site in the distal/proximal interphalangeal, metacarpo(tarso)phalangeal, radiocarpal or middle carpal joints. Lameness of minimum 4 weeks’ duration. Lameness positive to flexion test of the affected joint. Osteoarthritis confirmed by intra-articular anaesthesia and radiographic evidence (eg joint space narrowing, osteophytes and subchondral sclerosis). Signalment: Age: Median 11 years (range 3–23 years). Sex: Nine females, three stallions, and 16 geldings. Breed: Twelve Warmbloods, nine Quarter Horse / American Paint / Appaloosa type, five Thoroughbreds, one Haflinger, one Saddlebred. Use: Twenty sport horses, one racehorse, seven pleasure horses. Exclusion criteria: Intra-articular therapy of the affected joint within 4 weeks before enrolment or during the study period. Systemic application of CSs, non-steroidal anti-inflammatories, other pain medication, shock wave, acupuncture or any homeopathic or oral supplements within 4 weeks before study enrolment or during the study period. Horses that had surgery within 90 days of enrolment. Multiple joint lameness. Fractures. Grade 5 lameness. Pregnancy.
Sample size:	28 horses.
Intervention details:	All horses received 2.5 ml of 4% polyacrylamide gel (Noltrex, RC Bioform LLC, Moscow, Russia) by aseptic intra-articular injection. Horses then underwent an exercise protocol according to their use. Five horses were administered a second injection after day 45 (and were excluded). Clinical evaluation at enrolment, 21, 45, and 90 days.
Study design:	Prospective non-comparative study.
Outcome studied:	Subjective assessment. Variables were assessed using a scoring system: Lameness (AAEP scoring system). Flexion test response. Pain on manipulation. Range of motion. Swelling. Radiographic score (before enrolment) (van Hoogmoed et al., 2003). Primary outcome: clinical improvement at day 45 defined as: Reduction of at least one lameness grade and/or; Combined reduction of at least three among scores for pain, range of motion, and joint swelling. Secondary outcome: clinical improvement at day 90 as defined above.Horses removed from the study or that had additional interventions between days 45 and 90 were considered failures. Horses removed from the study or that had additional interventions between days 45 and 90 were considered failures.
Main Findings (relevant to PICO question)	23/28 (82%) success rate at day 45. Response to therapy predominantly occurred in the first three weeks after treatment. 21/28 (75%) success rate at day 90. Significant decrease in lameness score between day 0 and 45.
Limitations:	No control group. Investigators selected horses for the trial and were not blinded for evaluation, which may have led to bias. Subjective measurements were used. Measurements of success do not necessarily indicate soundness or a return to previous level of work. Restricting exercise post-treatment may have been confounding; exercise protocols varied according to the horses’ use. Limited follow-up period (90 days). Study funded by the exclusive distributor of 4% polyacrylamide in North America.

**de Clifford (2019):** 

Population:	Horses at a single training facility presenting for routine veterinary clinical examination for lameness between 1 June 2015 and 31 May 2016 with: Lameness originating from one or more joints (metacarpophalangeal, intercarpal, and radiocarpal) associated with clinical signs of joint inflammation (effusion, heat, swelling, pain). Lameness confirmed by a positive response to intra-articular analgesia (100 mg mepivacaine hydrochloride (Mepivacaine injection, Ceva Animal Health Pty Ltd, Glenorie, NSW, Australia) per joint, horses re-examined after 10 minutes). In horses with multiple joint lameness intra-articular analgesia was performed in a different sequence to determine the lameness grade in all affected joints. Radiographs ensuring no confounding factors. Signalment: Age: Mean 5 years (range 3–7 years). Breed: Thoroughbred. Use: Flat racing – working at gallop and racing distances of 1.2–2 km. Exclusion criteria: Osteochondral fragmentation or articular fracture. Joint lameness secondary to infection. Lameness grades of 4 and 5 (modified AAEP lameness scale). Horses having undergone surgery of the joint 3 months preceding or during the study. Any anti-arthritic intra-articular treatment involving the affected joint(s) 2 months preceding or during the study. Use of systemic nutraceuticals throughout the study. Systemic anti-inflammatories within 14 days of examination.
Sample size:	49 horses (89 joints).
Intervention details:	Day 0: All horses received aseptic intra-articular injection in the affected joint(s) with 2 ml of 2.5% polyacrylamide gel ((PAAG) Arthramid Vet; Contura International A/S, Soborg, Denmark) with 100 mg gentamicin (Gentamax 100, CEVA Animal Health, NSW, Australia). All horses were rested for 48 hours after treatment before re-entering training. Follow-up performed at 1, 4, 12, and 24 weeks post injection.
Study design:	Non-comparative pilot study.
Outcome studied:	Subjective assessment. Variables measured: Lameness score (modified AAEP scale). Radiological score (at baseline). Joint effusion score. Response to flexion test.
Main Findings (relevant to PICO question)	Significant improvement in lameness grades at weeks 1, 4, 12, and 24 compared to week 0. 0/49 (0%) of horses were free from lameness at 1 week, 21/49 (43%) at 4 weeks, 33/49 (67.3%) at 12 weeks and 32/49 (65.3%) at 24 weeks. 7/49 (14.3%) of horses were not free from lameness but improved enough to remain in race training. Largest reduction in lameness scores occurred at 4 weeks. No significant reduction in joint effusion score was seen.
Limitations:	No control group. Horses were selected without randomisation which may have led to bias. Some signalment details not included. Observer was not blinded. Horses with higher lameness grades were excluded which may have led to bias. 48/89 (54%) of horses had no radiographic signs of OA so may have had other joint pathology or early OA. Systemic anti-inflammatories were permitted during the study (although not within 14 days of examination). Variables measured were subjective. The study does not provide detail on horses that remained lame. Follow-up was limited to 6 months. Discrepancy in reporting lame-free horses at 24 weeks between the results and discussion sections. Conflict of interest declared by lead author.

**de Souza (2020):** 

Population:	Adult equines of any breed and modality, with or without joint disease.
Sample size:	19 papers were included for qualitative assessment. These involved 3–30 horses (mean 11). One study involved polyacrylamide gel (PAAG).
Intervention details:	The included studies Investigated intra-articular use of hyaluronic acid (HA) or PAAG for the treatment of equine OA. Compared the above medications with a placebo and/or other medication. Evaluated the in vitro or in vivo effect of intra-articular HA or PAAG in normal or diseased tissues.
Study design:	Systematic review.
Outcome studied:	Subjective assessment – a meta-analysis was not possible due to the heterogeneity of included studies. The authors investigated whether evidence from the scientific literature supports or refutes the use of HA and PAAG as intra-articular therapy in equines.
Main Findings (relevant to PICO question)	One study examined PAAG; the safety of administration in healthy joints: 5 ml 4% PAAG was used. There was no evidence supporting clinical efficacy of PAAG therapy in equine OA.
Limitations:	No explicit PICO question was asked in this study. In the search strategy only one term for PAAG was used when other variants are possible. This could have led to titles being excluded. The term ‘osteoarthritis’ was not included in the search strategy. Non-randomised and in vitro studies were included. The methodological quality of included studies was not assessed.

**Greene (2020):** 

Population:	Horses lame due to OA of the distal tarsal joints.
Sample size:	Three papers: Two randomised controlled trials. One retrospective study.
Intervention details:	The included studies: Investigated use of either intra-articular CS or a systemic bisphosphonate as a therapeutic intervention. Investigated horses with lameness due to OA of the distal tarsal joints.
Study design:	Systematic review.
Outcome studied:	The authors investigated whether intra-articular CS is more effective than systemic bisphosphonate treatment in long-term lameness reduction in horses that are lame due to distal tarsal joint OA.
Main Findings (relevant to PICO question)	One study reported a positive correlation between treatment with intra-articular CSs for distal hock OA and a modest improved outcome: This paper provided the strongest evidence to support intra-articular CSs as the sole treatment in chronic OA. One controlled trial investigating systemic bisphosphonate, although finding significantly lower lameness scores in the treatment group, had poor quality evidence; change to clinical practice was not recommended on this basis. Long-term outcome of intra-articular triamcinolone treatment is not positive for distal tarsal OA. There is insufficient evidence to support use of systemic bisphosphonates over intra-articular CSs to treat equine distal hock OA.
Limitations:	The paper described as the strongest evidence for CSs did not specify the numbers of horses which also received other intra-articular treatment (hyaluronic acid). This weakens the evidence for CSs. The same paper was excluded from this Knowledge Summary for this reason. Two papers reviewed did not use CS or bisphosphonate as a single treatment. Other treatments that could have affected lameness were used in the study protocol, e.g. hyaluronic acid, non-steroidal anti-inflammatories. No process for paper assessment is described. No statistical analysis possible due to the variability of the papers.

**da Silva Xavier (2021):** 

Population:	Horses with OA – all included articles evaluated horses with at least one joint affected by degenerative disease confirmed by clinical and complementary (for example, radiographic) examination.
Sample size:	23 papers were included for systematic review. 16/23 papers were included in the network meta-analysis. 6/23 papers involved polyacrylamide gel (PAAG) – all six were used in the systematic review and meta-analysis. 724 horses were evaluated in included studies. 188 horses were in studies involving PAAG. 160 horses received a single intra-articular PAAG application.
Intervention details:	The included studies: Had an observational and / or experimental design. Used PAAG or hyaluronic acid (HA) (alone or in combination with other substances). Compared the degree of lameness before and after treatment. Used resolution of lameness as an outcome. Studies included in the meta-analysis: Described the intervention clearly. Used a validated outcome measure. Used a prospective design. Clearly presented the results regarding animals that were removed or lost from the study.
Study design:	Systematic review and meta-analysis.
Outcome studied:	The efficacy of PAAG alone or HA alone and combined with other products and in reducing lameness in horses with OA.
Main Findings (relevant to PICO question)	The probability of lameness being reduced was highest with PAAG followed by HA and HA combined with other treatments. The reduction in lameness associated with PAAG was significantly greater compared to HA alone or combined with other products. 120/160 (75%) horses treated with a single intra-articular application of PAAG had relief from pain and lameness. No adverse effects were associated with PAAG. PAAG has a long duration of action.
Limitations:	Studies with a control group for comparison were not required in the inclusion criteria. In some HA studies a control group, involving the application of saline, was used; no horses showed improvement in clinical signs. From this, the authors state that it is possible to determine the efficacy of a drug without a control group as ‘it is known there will never be a spontaneous improvement’. Most studies were not randomised. No study involving PAAG used a control group. All PAAG papers involved selection bias and detection bias. Only indirect comparisons between studies could be made. All studies showed a moderate risk of bias for PAAG and control comparisons.

## Appraisal, application and reflection

There is currently no evidence to support the use of intra-articular PAAG compared to CS to alleviate symptoms of lameness associated with OA in horses. To reach this conclusion, papers investigating the use of each treatment for OA were appraised and, in general, the strength of evidence was weak because most studies did not involve a control population. Direct comparison of the studies reviewed was inappropriate due to differing sample sizes and methodology. No single paper compared the response to treatment using PAAG or CS alone. Unfortunately, the most relevant clinical study documenting significant difference had to be excluded as the comparison was made between PAAG and combined hyaluronic acid and CS (Tnibar et al., 2014b).

Anecdotally, steroids are effective in alleviating lameness caused by OA; this is supported by research involving horses with induced OA. A significant reduction in lameness has been demonstrated following injection of triamcinolone and 6-alpha-methylprednisolone acetate in an osteochondral fragment model (Frisbie et al*.*, 1997; and Frisbie et al*.*, 1998). Triamcinolone acetonide has produced significant analgesia in lipopolysaccharide-induced synovitis (Kay et al., 2008; and Neuenschwander et al., 2019). A review of the literature reporting the use of CS in models of OA is beyond the scope of this Knowledge Summary.

Pain relief has been attributed to the inhibition of prostaglandin synthesis through inhibition of the enzyme phospholipase A2 and reduction of cyclooxygenase ((COX)-2)) expression (Caron, 2005). Multiple effects on signal transduction pathways are reported in the medical literature, with upregulation of anti-inflammatory genes that produce molecules such as interleukin-10 and inhibitor of nuclear factor kappa-beta (NF-kB) and suppression of genes that code for inflammatory proteins through inhibition of pro-inflammatory transcription factors such as NF-kB (Barnes, 2006). Evidence for intra-articular corticosteroid consisted largely of case series (van Pelt 1963; van Pelt et al., 1970; and van Pelt et al., 1971) with poor methodology that compromised the interpretation of their findings. The studies reported the use of 6-alpha-methylprednisolone, betamethasone disodium phosphate, betamethasone acetate, and flumethasone. In these studies, samples were small, and diagnoses were often vague with no mention of diagnostic anaethesia in the lameness investigation. Clinical outcomes were often reported as secondary data to synovial fluid analysis. Reporting of clinical outcomes was extremely subjective with little or no information on lameness severity prior to treatment and no attempt to grade the lameness using a scale. There was also limited or no information regarding follow-up or whether the horses returned to their previous level of activity. In the study by Nicpoń et al. (2013), CS was not the focus of the research, but provided a comparison with the use of intra-articular mesenchymal stem cells. The evidence was of poor quality due to a small sample size, a lack of detail in the methodology, no diagnostic imaging, no lameness grading, and no numerical data or statistical analysis. The study by de Grauw et al. (2016) was of better quality with use of a lameness scale (Ross, 2003) and randomisation of treatments; however, there was no diagnostic imaging used as an inclusion criterion, no blinding and follow-up was limited to 3 months. The latter two studies were included despite a lack of radiographic evidence to confirm OA as they were considered pertinent to the PICO question and given the high prevalence of OA as a cause of equine articular pathology (Frisbie, 2006; Neundorf et al., 2010; and de Souza, 2016). The systematic review by Greene (2020) involved a thorough literature search and indicated moderate evidence for the use of CS to treat distal tarsal OA; however, any alleviation of lameness was not long term. Similar to the present Knowledge Summary, Greene (2020) was limited by the lack of controlled studies. Another significant weakness was that the selected papers involved the combination of CS with other anti-inflammatory agents.

The lack of good-quality evidence using controlled trials to investigate intra-articular CS in clinical OA is surprising given that this is extremely common practice in equine orthopaedics. In a survey conducted by the American Association of Equine Practitioners, 549/737 (74.5%) of respondents used intra-articular corticosteroid in joints with radiographic evidence of OA. A majority (621/803 [77.3%]) used triamcinolone acetonide in high-motion joints and (584/803 [72.7%]) used methylprednisolone acetate in low-motion joints (Ferris et al., 2011). From the evidence analysed in this Knowledge Summary, it can be inferred that CSs do have an effect in reducing lameness due to OA but that this effect is likely short term.

Polyacrylamide gel is a synthetic non-degradable, biocompatible and non-toxic substance with water-exchanging abilities (Zarini et al., 2004; and Brahm et al., 2012). It is a relatively recent addition to the clinician’s options when considering treatments for OA. Christensen et al. (2016a) demonstrated integration of PAAG into the synovial layer in normal and osteoarthritic equine joints examined post-mortem up to 2 years post injection. Similar changes were demonstrated in normal rabbit knee joints up to 1 year post injection.  Mechanisms by which PAAG may alleviate the clinical signs of OA are currently unknown. Its viscoelastic properties may have a shock absorbing effect, reducing impact forces on joints during exercise (Janssen et al., 2012; and da Silva Xavier, 2021). Another hypothesis is that joints with PAAG have better elastance compared to control joints (Tnibar et al., 2014a; and Tnibar et al., 2017); human knees affected by OA have a greater stiffness coefficient compared to normal controls (Hall et al., 2014). Furthermore, horses with OA that responded well to PAAG showed a reduced pain response to passive joint manipulation (Tnibar et al., 2015). Alternatively, PAAG may cover subchondral mechanoreceptors, preventing neural stimulation (Janssen et al., 2012); reduced mechanoreceptor activation may also occur due to increased elasticity of the joint (de Clifford et al., 2019). Coating the cartilage surface may also facilitate fibrocartilaginous healing (Slesarenko & Shirokova, 2012). Polyacrylamide gel may provide viscosupplementation and aid joint lubrication (Tnibar et al., 2014a; and McClure & Wang, 2017) which in turn could reduce pain (Silva Cabete, 2018). Viscosupplementation may also aid disease stabilisation (Tnibar et al., 2014a; and Tnibar et al., 2017).

In this Knowledge Summary, there were no controlled studies investigating PAAG in naturally occurring equine OA. This weakens the rationale for its use. Studies involving PAAG to treat OA were prospective and non-comparative. Overall, the methodology and reporting in these studies were better than those reporting CS use, perhaps due to their more recent publication. Limitations often included a small sample size (12–49 horses), subjective assessment of lameness, potential selection bias and a lack of blinding. One systematic review (de Souza et al., 2020) investigated the use of PAAG but its conclusions were similarly limited by the lack of controlled trials. Although a meta-analysis is considered the highest level of evidence (Murad et al., 2016), the study appraised in this Knowledge Summary (da Silva Xavier et al., 2021) was limited by a lack of controlled trials and bias, and the heterogenous nature of the studies weakened the statistical analysis that could be performed. The authors stated that it is possible to determine the efficacy of a drug without a control group as ‘it is known there will never be a spontaneous improvement’ – this was due to some horses showing no improvement after application of saline in studies relating to hyaluronic acid (HA). This, however, seems a tenuous assumption.

One comparative study (Tnibar et al., 2014b) reported significant improvement in horses with OA treated with PAAG compared to those treated with triamcinolone acetonide and hyaluronic acid. The addition of hyaluronic acid to intra-articular steroid meant that this paper was excluded from the present analysis. Several papers have considered horses to act as their own controls however this is not ideal. The abstract by Bathe et al. (2016) was excluded from this Knowledge Summary due to its being a conference proceeding. The authors reported a case series of 20 horses with lameness due to OA of the proximal or distal interphalangeal joints diagnosed using diagnostic anaesthesia and imaging (radiography or standing MRI). All horses had not responded to previous intra-articular CS injection. All horses received 1 ml of PAAG (ArthramidVet, Contura International A/S, DK-2860 Søborg, Denmark) then underwent 4 weeks of exercise restriction before a progressive return to ridden exercise. Re-examination and a telephone survey were used to follow-up the cases; median follow-up time was 12 months. The average duration of lameness was 15 months and the average lameness score was 3/10 (grading system not given). One horse was treated twice and had a transient adverse reaction (not fully described). 12/18 cases returned to full function, 3/18 to lower level function and 3/18 failed to improve (two horses were lost to long-term follow-up). Although these cases had previously failed to respond to intra-articular corticosteroid, a direct comparison between horses receiving corticosteroid and PAAG would provide more robust evidence for intra-articular PAAG to treat OA. In this Knowledge Summary,  Janssen et al. (2012) reported a small case series of horses that responded to PAAG which had chronic or recurrent lameness for greater than 3 months’ duration due to distal interphalangeal joint OA diagnosed using nerve blocks, intra-articular anaesthesia, radiological and MRI findings. The horses had previously failed to respond to intra-articular glucocorticoid, hyaluronic acid and autologous conditioned serum, used alone or in combination; however exact treatment protocols and timings were not described. De Clifford et al. (2019) reported that 20% of horses had failed to respond to other treatments.  The authors suggested that these horses could have been considered as their own positive controls; however, small case numbers and lack of detailed treatment information precluded statistical analysis. Similarly, Tnibar et al. (2015) reported that 86% of horses had received prior unsuccessful anti-osteoarthritic treatment which, again, was not defined in detail. Retrospective analysis is not ideal, and a control group is required for meaningful assessment.

In an experimental OA model, 3/4 of goats were non-lame 4 months after treatment with PAAG; 2 control goats remained lame (Tnibar et al., 2014a). Whilst used in therapeutic and cosmetic surgical augmentation of soft tissue structures (Fernández-Cossío & Castaño-Oreja, 2006; Bello et al., 2007; Christensen, 2009; Pallua & Wolter*,* 2010; and Sokol et al., 2014) and ocular bioimplants (Lloyd et al., 2001), PAAG has also been used in the treatment of human-knee OA (Zar et al., 2012; Christensen & Daugaard, 2016b; Issin et al., 2018; Henriksen et al., 2018; and Overgaard Sr et al., 2018). Comparative and controlled studies are similarly limited. Issin et al. (2018) compared PAAG with methylprednisolone for patients with knee OA and found no statistically significant difference between the treatments based on the Western Ontario and McMaster Universities Arthritis (WOMAC) Index and patient self-assessment. A limitation of this study was the heterogeneity of the treatment groups. In a prospective cohort study, authors found statistically and clinically significant reductions in WOMAC pain, WOMAC stiffness, physical function, and WOMAC total after 4 months which persisted after 1 year. No control group was included in this study and subjects were not blinded (Henriksen et al., 2018). The evidence assessed in this Knowledge Summary indicates that intra-articular PAAG may be effective in alleviating lameness due to naturally occurring OA in horses and that its effect may be longer term than CSs. One therapeutic agent cannot be recommended in favour of the other as there are no experimental studies which compare them.

Osteoarthritis is a frequent cause of equine lameness hence this PICO question is extremely relevant to clinical practice. To answer this question effectively, a prospective randomised, blinded control trial including a large sample size of horses with naturally occurring OA with considerable long-term follow-up is warranted. Suggested inclusion criteria for such a study would be horses with OA diagnosed using diagnostic anaethesia and a thorough imaging assessment including radiography and MRI; a detailed, standardised anamnesis and clinical examination should also be performed. Horses that received any other potentially lameness-altering medication prior to or during the study should be excluded. Corticosteroid and PAAG should be trialled in isolation (in this Knowledge Summary, studies were excluded if the therapeutic agents of interest were used in combination with other medication that could alter lameness). A non-treated control group would be ideal; however, this is not ethical in a lame animal. In addition, some horse owners would be unlikely to consent to such a study unless their horse received some form of treatment. Objective gait analysis, to eliminate observer bias, is also recommended (Keegan et al., 2010). Suitable outcomes that could be measured in addition to lameness severity could include return to previous level or a higher level of work, joint circumference and owner satisfaction. Standardisation of outcomes would facilitate the development of meta-analyses. With this information, more meaningful conclusions regarding the optimal treatment for clinical OA in equines could be made.

## Methodology Section

**Search Strategy:** 

Databases searched and dates covered:	CAB Abstracts on OVID interface 1973–2021 week 31 PubMed on the NCBI interface from 1920 to August 2021
Search strategy:	CAB Abstracts: horse* or pony or ponies or equine* or exp horses/ osteoarthrit* or osteo-arthrit* or OA or 'joint disease*' or DJD or arthrit* or exp osteoarthritis/ or exp arthritis/ or exp joint diseases/ polyacrylamide or arthramid or aquamid or PAAG steroid* or corticosteroid* or glucocorticoid* or corticoid or triamcinolone or triamcinalone or methylprednisolon* or prednisolon* or exp glucocorticoids/ or exp prednisolone/ or exp prednisone/ or exp steroids/ 1 and 2 and (3 or 4) PubMed: horse or pony or ponies or equine arthritis or arthritic or arthritogenic or arthritical or osteoarthritis or osteoarthritic or osteo-arthritis or osteo-arthritic or "joint disease" or "joint diseases" or OA or DJD polyacrylamide or arthramid or aquamid or PAAG steroid or corticosteroid or glucocorticoid or corticoid or triamcinolone or triamcinalone or methylprednisolone or prednisolone 1 and 2 and (3 or 4)
Dates searches performed:	02 Aug 2021

**Exclusion / Inclusion Criteria:** 

Exclusion:	Not relevant to question including in vitro studies and induced synovitis. Use of combination treatments where effect of either therapeutic agent alone is not explicit. Non-English language. Narrative review, conference proceeding, opinion article, book chapter. Papers that were not available.
Inclusion:	Use of CSs and or PAAG to treat OA in the horse where clinical signs associated with lameness were measured as an outcome.

**Search Outcome:** 

Database	Number of results	Excluded – Irrelevant to question	Excluded – Relevant but non-English language	Excluded – Relevant but non-systematic review, conference proceeding, book chapter	Excluded – Relevant but not available	Total relevant papers
CAB Abstracts	385	282	25	62	7	9
PubMed	205	181	2	16	0	6
Total relevant papers when duplicates removed						12

## Conflict of Interest

The authors declare no conflicts of interest.

## Disclaimer

Knowledge Summaries are a peer-reviewed article type which aims to answer a clinical question based on the best available current evidence. It does not override the responsibility of the practitioner. Informed decisions should be made by considering such factors as individual clinical expertise and judgement along with patient’s circumstances and owners’ values. Knowledge Summaries are a resource to help inform and any opinions expressed within the Knowledge Summaries are the author’s own and do not necessarily reflect the view of the RCVS Knowledge. Authors are responsible for the accuracy of the content. While the Editor and Publisher believe that all content herein are in accord with current recommendations and practice at the time of publication, they accept no legal responsibility for any errors or omissions, and make no warranty, express or implied, with respect to material contained within. For further information please refer to our Terms of Use.
